# Women and nature: Ecofeminist study in social media

**DOI:** 10.12688/f1000research.162436.1

**Published:** 2025-04-23

**Authors:** Jhilik Chakraborty, Arpita Goswami

**Affiliations:** 1 Department of Humanities, School of Liberal Studies, Kalinga Institute of Industrial Technology (KIIT) Deemed to be University, Bhubaneswar, Odisha, 751024, India; 2 Department of Humanities, School of Liberal Studies, Kalinga Institute of Industrial Technology (KIIT) Deemed to be University, Bhubaneswar, Odisha, 751024, India

**Keywords:** Ecofeminism, Women, Nature, Digital Platform, Social media, Facebook, Instagram, Youtube, Representation

## Abstract

**Background:**

Social media has turned into an important way for ecofeminists to raise awareness, create supportive communities, and push for fairness in environmental matters. Women activists, influencers, and groups are using social media sites like Instagram, Facebook, and YouTube to bring attention to topics like climate change, sustainability, and the link between gender and environmental issues.

**Methods:**

By taking Ecofeminism as a theory, the paper examines selected accounts from three different social media platforms viz, Facebook, Instagram, and YouTube to explore how they reflect the theme of ecofeminism. Ecofeminism is a theory that highlights the connection between women and nature, advocating for their protection.

**Results:**

Our findings establish that the principles of ecofeminist theory and the works of ecofeminists have moved beyond physical propagation and are now being actively disseminated on digital media platforms. Through various forms of media such as reels, videos, pictures, and animations, many social media accounts vividly demonstrate the close relationship between women and nature, the exploitation they face from patriarchal society and how they can be protected.

**Conclusion:**

Since the people of the current age are highly engaged with social media, these platforms effectively communicate the concept of ecofeminism and its importance, motivating global audiences to treat, protect, and preserve both nature and women. This helps in creating an inclusive society and advancing a sustainable world, which is critical in today’s context of environmental degradation and social unrest. The paper contributes to the fields of ecofeminism, media studies, and the sustainable development goals established by the UN in 2015.

## Introduction

In contemporary society, both the environment and women are facing significant challenges, such as environmental degradation and women’s exploitation. In a country like India, women are revered and given the status of Goddesses. However, despite this cultural respect, women continue to face numerous issues that starkly contradict this ideal. Almost every day, women face some form of violence like domestic assault, sexual misconduct, marital rape, and dowry-related issues. Discrimination against women is another significant issue. In our society, discrimination between men and women exists in many respects, such as women receiving lower pay for the same work, facing higher rates of domestic violence, and having fewer opportunities for leadership roles in corporate and political spheres. On the other hand, environmental degradation occurs because of many factors such as growing populations, urbanization, energy use, and transportation. Various theories aim to address the exploitation of women and environmental degradation, such as environmentalism, ecofeminism, ecocriticism, and feminism. A movement of concerned citizens, environmentalism aims to preserve the environment in which we live. A major goal of environmentalism’s early development in the late 1800s was to increase public awareness of and appreciation for nature’s beauty. Environmentalism is a movement focused on protecting and enhancing our natural world. It encourages changes in the way people act that negatively affect the environment. This includes creating political, economic, and social systems that help people treat nature with care, as well as reevaluating our relationship with it. Environmentalism is the belief that determines the morality of political, economic and social policies. Feminism advocates for dismantling gender inequality by promoting the equality of all genders. It addresses issues such as pay inequality, access to healthcare, rigid social expectations, and gender-based inequalities. Ecofeminism combines feminism and environmentalism, positing that both the degradation] of the environment and the oppression of women stem from capitalism and patriarchy. Feminists explore the reasons behind the exploitation of women, while environmentalists explore the reason behind the exploitation of the natural world. The ecofeminists identify patriarchal development attitudes as the common source behind the mistreatment of both women and nature. Therefore, ecofeminists consider the powerful gender, typically men, to be exploiters of both women and nature.

Let’s talk about digital platforms. A digital platform refers to a space where buyers and sellers come together to exchange information, products, or services, along with the community of users who participate in this exchange ( Watts, 2020). It is a critical element to realise that the community is a digital platform; without the community, the platform wouldn’t be as valuable ( Watts, 2020). We are actively engaging with digital platforms. This is because media technologies influence every aspect of our lives, influencing human consciousness and the way we perceive the world around us as well as our socio-environments. Ecofeminism is an interdisciplinary movement that links the pursuit of environmental justice with the proliferation of gender equality, and digital platforms are essential to its advancement in many ways. Digital platforms serve as platforms for ecofeminist activists, in particular individuals from marginalized communities to garner the public as a voice to tell their stories, thoughts, and struggles. Social media provides a mechanism for grassroots activists to communicate to a global audience, raising awareness about local environmental problems and their gendered implications. Digital tools such as discussion boards, social media, and collaborative platforms allow ecofeminists to form online communities to connect, exchange resources. These communities promote solidarity across geographic boundaries, linking activists working on shared problems, such as climate change, deforestation or water rights. Digital tools help in providing information, research and campaigns on ecofeminism. Blogs, webinars and online courses explain to the public connections between environmental degradation and gender inequality. Digital platforms provide ecofeminists the ability to work with intersectionality across numerous other areas such as race and class and colonialism that would hardly be possible without digital spaces.

So, in short, digital platforms elevate ecofeminism, by amplifying voices, creating communities, spreading awareness, and stimulating action on a global scale. These tools are critical in tackling the institutionalized inequalities that sit at the centre of environmental and gender injustices. Thus, the results of this study are examined using social media platforms such as Instagram, Facebook, YouTube and ecofeminism theory.

There are some existing works which talked about how social media platforms and how they are propagated in feminism and ecofeminism. Many existing works in English literature explore how different literary texts raise the theme of ecofeminism. Griffin (1978) explores women’s roles and identifications with nature, as well as how they view the environment and human relationship to it using compelling language and exquisite prose and poetry. Griffin uses a wide range of sources- from ordinary medical guidelines to classical literature- to show how patriarchy has permeated every aspect of existence and to compel readers to reconsider language. Similarly, Plumwood (1993) explains the connection between the world’s current predicament and the idea that masculine dominance is equivalent to that of nature. Given the importance of these two ideas and the political movements that arose in the latter half of the 20th century, Plumwood contends that feminist theory has a significant chance to advance political ecology and environmental philosophy, which may have influenced the development of the current climate justice movement. Both the two books explore the theme of ecofeminism but they differ in style, approach, content. Plumwood’s work sets out to question and break down Western ways of thinking that separate humans from nature. It suggests a fresh perspective that sees how closely we are all linked. The aim is to create a base for ecofeminist ethics that opposes all kinds of oppression. On the other hand, Griffin wants to highlight the stories of women and nature that often go unheard, blending personal experiences with history and mythology. Her hope is to spark a strong emotional bond and foster an understanding of the struggles faced by both women and the environment. Plumwood provides a thoughtful way to look at how the struggles of women and nature are linked, while Griffin takes a more poetic and storytelling approach to these ideas. Together, they work well together; Plumwood sets up the theory, and Griffin brings in the feelings and creativity of ecofeminism.

Ruiz and Garcia (2021) examine feminist posts on Latin American Facebook pages that touch on the body and sexuality, two topics that have historically served as the foundation for oppressive language and laws. The study adds to the body of information regarding the dynamics of feminism in the digital age, which has succeeded in creating new possibilities for women in Latin America to exercise their freedom on an individual and shared level while also resisting patriarchal discourses. Moreover, Pruchniewska (2019) illustrates how private Facebook groups offer opportunities to create environments similar to second-wave consciousness-raising groups through interviews with 26 women. Apart from cisgender men, these groups allow women to talk about their professional experiences and exchange advice, which affects women even outside of the groups. Therefore, although not overtly feminist, private Facebook communities for working women support feminist ideals. Similarly, some works portray the concept of feminism on YouTube. For instance, Serrano-Contreras (2021) investigates how it has expanded on a website such as YouTube. In this paper, new computer techniques are explored to investigate the concept of feminism in the digital world. This study uses Natural Language Processing to explore how feminism has evolved on YouTube, especially in Spain. It shows a noticeable rise in conversations about Spanish feminism on the platform, especially after 2016, highlighting two different viewpoints in the discussion. This research helps us see how platforms like YouTube can be spaces for public conversations about important social topics like feminism.

However, there is a lack of significant works that highlight the promotion of ecofeminism through the digital world. Nevertheless, some works do emphasize the promotion of feminism in the digital realm. For instance, Swer (2019) has used a different kind of ecofeminism known as Transformative Ecofeminism which makes a connection between women and nature. Transformative ecofeminists contend that due to the links between women’s oppression, women’s liberation has to involve the liberation of nature and vice-versa. This paper further argues that ecofeminism includes important technological analysis and contains significant components of technological analysis elements that can be interpreted as carrying on a technological philosophy. In this paper, it is shown that Warren’s ecofeminism views technology from an instrumentalist point of view. Many see technology as a specific object.

Furthermore, Singh and Bali (2022) have stated the dynamic relationship between media, literature, and ecofeminism, exploring how these realms intersect to shape and disseminate ecofeminist ideas. The theory of ecofeminism is used to investigate the eco-gender imbalance on Instagram. By examining carefully, the effect of media on ecofeminist narratives, this paper helps to increase the complex understanding of how these powerful methods shape public insights and strengthen the ecofeminist movement.

Furthermore, Jain (2020) has argued the impact of digitisation on women’s movements, particularly in developing nations like India. It achieves this by trying to apply postcolonial and postmodern feminist perspectives to the analysis of modern cyberfeminism. The study also identifies the benefits and disadvantages of online activism. According to some feminists, the restrictive hierarchies of the global political economy are recreated in the online world. But others think of it as a chance to actively participate in various ecofeminist movements and also as a new beginning for international feminist socialising. Cyberfeminism expanded rapid feminist movements that saw women as an integrated, similar category where a single law could meet their requirements. Feminist groups and organisations should look into the cultural communities they belong to when promoting online rights. To identify and prevent online prejudice, public agencies, governments, and businesses should take the responsibility of creating laws and social structures. It’s critical to recognise that, despite its apparent novelty, online misogyny is defined as a long-lived issue that derives from gender-based ability and control structures. On one hand, Singh and Bali explore historical and contemporary views on ecofeminism and on the other hand, Jain explores mainly on digital ecofeminism. Singh and Bali (2022) mainly focus on theoretical and gender studies and Jain (2020) focuses on socio-political dynamics and digital technology.

In this paper, we have taken three social media platforms which are Instagram, Facebook, YouTube and we try to explore how through these three social media platforms the concept of the theory of ecofeminism is getting reflected or propagated and for that purpose.

## Methods

The data used in this study has been collected from publicly available Instagram, Facebook, and YouTube pages for the purpose of analyzing themes related to women and nature. We have collected data from these social media accounts based on themes, hashtags used, engagement metrics and audience interactions ensuring a comprehensive analysis of how ecofeminism is reflected and propagated on these platforms. This research follows a qualitative paradigm. The study seeks to understand the meanings and interpretations attached to ecofeminist themes within digital spaces, focusing on the subjective experiences of content creators and their audiences.

Due to ethical considerations, including the lack of consent from the original content creators, the data has been de-identified to ensure that no personally identifiable information is present. The researchers conducting this study have a strong interest in ecofeminism, media studies, and environmental issues. They approach the research with an objective mindset, focusing on analyzing social media content without including personal opinions or experiences. The researchers are aware of potential biases and ensure these do not influence the findings, striving to maintain objectivity throughout the analysis.

The sampling strategy includes purposive sampling of publicly available social media content that highlights ecofeminist themes, with an emphasis on posts, hashtags, comments, and videos. Given the wide range of global ecofeminist movements on digital platforms, content creators who identify as ecofeminist activists or advocates for environmental and gender justice are prioritized in the selection of materials. The data collection methods involves manual identification of relevant content. In terms of data analysis, the study applies qualitative content analysis methods, focusing on themes, language, and visual content in posts to understand how ecofeminism is communicated through social media. A mixed-methods approach is also be used, combining qualitative analysis with the assessment of engagement metrics such as likes, shares, comments, and followers to evaluate the impact and reach of ecofeminist content.

The ecofeminism theory is applied as the theoretical background to investigate the relationship between women, nature, and social media pages. Ecofeminism, an interdisciplinary ‘intellectual and political movement’ that gained prominence in the 1980s, represents a fusion of environment-friendly research and feminist ideologies. According to Sturgeon (1997), “Ecofeminism as a term indicates a double political intervention, of environmentalism into feminism and feminism into environmentalism” ( Sturgeon, 1997:169). Warren (1994) further elaborates that ecofeminism is “structurally pluralistic, rather than structurally reductionist or unitary: it emerges from a multiplicity of voices, especially women’s voices across cross-cultural contexts” (Warren, 1994:84). French author Françoise d. Eaubonne first used the word “ecofeminism” in her seminal work La Feminisme ou la mort in 1974. Over time, the term has evolved into a theory focused on practical application and is also referred to as quilt theory ( Patil, 2020). Scholars such as Vandana Shiva, Bina Agarwal, Carol J. Adams, Ynestra King, Maria Mies, and Greta Gaard have advocated for the concept of the interrelationship between nature and women, emphasizing the interconnectedness of various forms of oppression, such as the exploitation of women, nature, and animals within patriarchal systems. Important works like Staying Alive: Women, Ecology, and Development ( Shiva,1988), Ecofeminism ( Mies and Shiva, 1993), and The Sexual Politics of Meat ( Adams, 1990) focus on these critical interconnections.

Ecofeminism is a fusion of two words, ‘ecology’ and ‘feminism’. Ecology refers to the scientific study of living organisms and their interactions within the natural environment. Feminist movements, on the other hand, aim to eradicate gender inequality and empower women. While environmentalists focus on the exploitation of the natural world, feminists focus on the exploitation of women. Ecofeminists argue that patriarchal development attitudes are the common root behind the mistreatment of both women and nature, positioning men as the primary exploiters of both ( Gaard, 2015). The central objective of these movements is to liberate both women and the environment from the control of patriarchal power structures ( Patil, 2020). The study draws on the insights of influential ecofeminist scholars, such as D’eaubonne (1974), Ruether (1995), Merchant (1990), Mies and Shiva (1993), and Warren (1997). Patil (2020) highlights three key tenets of ecofeminism that guide this research: “(1) there is a connection between women and nature, based on (a) women’s close association with nature due to their dependency on it, and (b) the development of science and technology being the cause of the exploitation of women as well as nature. (2) Women are life givers, saviors, and nurturers of nature. (3) Patriarchy, under the guise of development, is the root cause of the exploitation of women and degradation of nature” ( Patil, 2020:32).

In alignment with these tenets, the study seeks to understand how ecofeminist themes are conveyed through social media platforms, with a focus on the connections between gender inequality and environmental degradation. It investigates how content creators within ecofeminist movements use these platforms to raise awareness, create communities, and amplify voices, contributing to the digital evolution of ecofeminism.

## Results and Discussion

The names of some of the well-known digital platforms nowadays are Facebook, Instagram, YouTube, LinkedIn, Twitter etc. In particular, this research explores the analysis of ecofeminism that mainly focuses on Instagram, Facebook and YouTube posts. Instagram is a popularly used social media sites that emphasizes distributing videos and photos. Similar to Facebook, Instagram allows anybody to make an account and includes a news feed, profile and other social media platforms ( Moreau, 2024). Instagram, which highlights mobile use and visual sharing, is comparable to a graded version of Facebook ( Moreau, 2024). Similar to other social networks, one can interact with other users by liking, commenting, tagging, following, and sending personal messages ( Moreau, 2024). On Android-powered phones and tablets from Google, Samsung, and other manufacturers, as well as iOS devices like the iPhone and iPad, Instagram is accessible for free ( Moreau, 2024). Finding and sharing the greatest images and videos is the primary goal on Instagram. Following and follower counts are presented on each user profile, manifesting the number of people they are following as well as the number of users who are following them ( Moreau, 2024). Certain well-known people with considerable online communities on social media or the internet at large, who frequently rely on their online presence for their livelihood, are considered Instagram influencers. Because Instagram is a popular platform for influencers, a lot of them are Instagram influencers ( Moreau, 2024). To investigate the notion of ecofeminism, this paper uses a few pages featuring female Instagram influencers who have a deep connection to the natural world.

Many Instagram pages are related to women in particular or nature individually. However, this study focuses on how ecofeminist ideas are discussed and shared on digital platforms such as social media posts, blog articles, videos, reels etc. The paper finds out how different digital posts on Instagram, Facebook and YouTube reflect ecofeminism theory. The findings of the paper show that some women on these social medias show their infinite love for nature through their posts. These women visit different places as travellers post their photos on Instagram, Facebook and YouTube and share their thoughts and love about nature.

By analyzing news articles, reports, and interviews, we can discern how news media contribute to the visibility of ecofeminism and whether these platforms effectively amplify the interconnected narratives of gender and the environment. The examination of news coverage related to climate activism focuses on how the media portrays women activists and their ecofeminist perspectives. Instagram wields a unique power to visually convey complex issues, making them instrumental in fostering ecofeminist understanding. Through the lens of different users and profiles, ecofeminist narratives can be brought to life, highlighting the stories of women at the forefront of environmental movements.

It is said that boys are taught to dominate and that girls are taught to stay domitable. Women feel that they pay a price to stay closer to the natural world. A woman feels mostly connected to nature when a resource is peeled off from nature because women too feel ignored and used. Women who face violence on their bodies mainly understand the pain done to nature. The Instagram page is a personal blog that is created to remind women to take care and stay closer to nature. There are few hashtags used in this page such as #womenlovenature 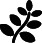, #bathnector and the engagement metrics of this page is 10.82%. This page shows that women are the most connected to nature. And while spending time with nature, woman can live freely in this world, she can make individual choices.

Another page taken for this research shows that a woman can live freely in this world, she can make individual choices. A picture in the page suggests that a woman should follow her dreams and stay positive. It is shown that nature cures a woman and that is why women are the most who have a magical, non-rational connection with nature. The idea of ‘magic’ is very important for some ecofeminists because nature offers mystical and magical moods that the culture-centered world cannot provide. The audience or followers are giving a positive feedback to the posts with words such as BEAUTIFUL, STUNNING, PURE BLISS etc.

Another Instagram page is about a girl and her dog who roams around the woodland and wonders in the northeast. The spectacular views and seasonal shots show how she is connected with nature. In one of the quote, a deep connection with nature is expressed, highlighting that the speaker feels most peaceful when she is travelling outside. She particularly cherish the beauty of the forests and enjoy observing the subtle, daily changes in the natural world. The hashtags used on this page are #scotland#forestphotography#neverstopexploring#welivetoexplore#exploremore#athomeoutdoors#naturelovers#forestlovers#thisisscotland#queenelizabethforestpark#forestryscotland#autumncolours#forestlovers# forestwalk#welovescotland and the engagement metrics of this page is 4.09%. All the comments by the followers are about the positive effects of spending time with nature. e.g. comments are beautiful capture, have a wonderful weekend, Super cool ride and shot beautiful work. The girl in a specific picture suggests that a woman should never stop exploring nature. She portrays through this image that she loved the feeling of being able to get lost and immersed in nature in the forest. While spending time in the forest, she feels that nature possesses the same qualities that a woman has. Women are seen as gentle, kind, domestic, beautiful, simple, graceful whereas men are seen as hard working, independent, industrial, assertive. Thus, according to the girl who owns this page thinks that nature embodies the same characteristics that women occupy. Gilpin quotes:

“Nature is always great in design. She is an admirable colorist also, and harmonizes tints with infinite variety and beauty.”

( Gilpin, *Observations on the River Wye* 1991)

Another well-known Instagram page is ideal for people who yearn to be in touch with nature. The girl in the page travels around the world wearing a red raincoat. For example, in one post she highlights her connection with nature. The girl portrayed in this page mentions in one of her quotes that she take photos to express her deep appreciation and reverence for nature, capturing its beauty and the emotions she feels while she is immersed in it. A Landscape & Travel Photographer shares her experience of travelling around the world and shows her love for nature through her writings. The hashtags used in this page are #paradiseart #timetobehave #nowherediary #ifyouleavestagram #somewheremag #subjectivelyobjective #artclassified #conceptmagazine #reframedmag and the engagement metrics is 0.531%. The Instagram feed is ideal for people who yearn to be in touch with nature. She travels around the world wearing a red raincoat. Her posts reflect her connection with nature. Comments like beautiful, gorgeous views and classy tips show how beautifully she clicked the pictures of nature.

Another Instagram page is based on an amazing storyteller with a vivid imagination. She produces a mythical and folkloric atmosphere through poetic captions that blend with whimsical images. Thus, in one of her posts she says that the speaker finds peace and inspiration in nature, seeing it as both a source of light and a gateway to magical, storybook worlds. The forest, with its wildflowers and shifting canopy light, becomes a comforting, enchanting refuge. The girl shares posts and reels on Instagram through her storytelling and vivid imagination. She produces a mythical and folkloric atmosphere through poetic captions that blend with whimsical images. There are less hashtags used in this such as #hobbithearted. The engagement metrics is 0.8115%.

Furthermore, there is another Instagram page which is about a girl who is an avid traveler and campaigner, particularly across the United Kingdom. She lives in a van and uses visual storytelling to communicate her connection to nature. Comments such as good idea, lovely storytelling, how amazing, full of wonders, would love to visit all of them highlight how followers take interest in seeing her travelling stories with an engagement metrics of 4.29%. The hashtags used in this page are #NaturalLiving #SustainableLiving #ConnectWithNature #SelfSufficiency #SeasonalLiving, #NatureLovers. She sets off on a quest to rewild herself since she has an obsession with nature. She uses folklore to describe her travels and the summer and winter seasons. In one of her posts, she describes how she believes that the summertime represents happiness, plenty, and a strong bond with the planet. The world is full of possibilities, the sun is at its highest point in the sky, and people are gathered to enjoy the longest day. The air is laden with the scent of blossoms. She claims that the fleeting nature of summer serves as a reminder of impermanence and inspires her to make the most of these valuable summer months. Now is the moment to act, follow her dreams and ambitions, and get in touch with like-minded individuals.

Furthermore, another page is about a girl who is a climate activist and fashion designer, makes a relationship between women’s attire and the environment. Her research is based on various fashion brands who are not supporting sustainable fashion. In some of her posts, she explains how today’s generation’s fashion business is based on monetary growth, with no ethical and environmental impact. Her posts reflect a transformation of the fashion industry with the help of everyone- consumers, brands, industry leaders and governments- to support sustainable fashion. The hashtags used in this page are #LoveYourNature, #SustainableLiving, #Earthday. The engagement metrics of this page is 79.73%. There are comments which appreciates her clear critique of the fashion industry and her focus on practical solutions. Another comment where people are thanking for sharing information suggest that the followers are very impressed with the knowledge shared by the girl in the profile. Her understanding of maintaining a balance between environment and sustainable living teaches the audience about how to maintain the relationship between environment and living.

In addition to talking about environmental protection, several Instagram accounts promote sustainability, youth and women’s empowerment, conservation, and the green narrative. Actually, the primarily female activist-run websites addresses younger female audiences, encouraging them to adopt a more active lifestyle and utilize more ethical and ecological products. For example, skin minimalism aims to decrease the use of skin care cosmetics and promote the reprocessing of used clothing by working with another (initiative in June 2022). Furthermore, as a component of their Instagram campaign for a green story, female protestors typically advocate ethical products and the hard work of women in micro industries in order to promote local and sustainable goods. Within environmental movements, female activists present themselves as social agents who, by working to overcome obstacles and offering suggestions for sustainable living practices and environmental laws, inspire society to take on more environmental responsibilities. This is consistent with the theory of ecofeminism, which upholds the idea that women may be change agents for environmental sustainability ( Resurrección, 2013). An individual who actively participates in post-sharing on Instagram is typically referred to as an enviro-fluencer, which is a person who encourages and persuades their viewers to take part in activism and certain causes. Environmental influencers are those who promote content related to climate change and sustainability, which is typically linked to increased offline interaction and encouraging sustainable behaviors ( San Cornelio et al., 2021; Dekoninck and Schmuck, 2022).

Social Media Platforms supplies an opportunity for detailed findings to reach out to various populations. Particularly, Facebook is the most ruling application in the social media panorama. During the past decades, there has been a growth of Facebook users between 145 million in 2008 to 1.2 billion in 2018 ( Desilver, 2014). Accordingly to reports, roughly two-thirds of the US population use Facebook ( Gramlich, 2019). Additionally, there are approximately 75% of Facebook users who browse Facebook daily and spend at least fifty minutes per day ( Gramlich, 2019) just to get some entertainment, read news, communicate with friends and family and exchange social support. Now, by taking a few Facebook pages, we will relate how the ecofeminism theory has been portrayed in those pages.

The first page which is taken to analyse the impact of ecofeminism in Facebook is a non-profit organisation created on 31st December 2023. This page aims to work towards equality between men and women and a green youth and encourages the social incorporation of women and girls through an union with sustainable exercises and advancing climate justice. The first point which comes in gender inequality is putting up barriers in women’s participation in various events and movements. We live in a society that tends to promote male superiority of action, decisions and choice. The engagement metrics of this page is 13.75% and the hashtags mainly used in this page are **#ecofeminism** **#project** **#green** **#equality** **#youth** **#youthwork** .

Marina explains the concept of ecofeminism by developing initiatives and strategies that support the sympathies and apprehension needed to build an inclusive culture that supports differences. Her efforts create a scope where everyone feels authorised to chase their dreams.

Another page is a non-binary gender identity created in Dublin on 14th April, 2019. This page offers research, training, education and consultancy services. One of the influencer in Facebook expresses that open and honest communication is necessary for understanding, resolving conflicts, and building relationships—it’s not something extra or optional. It’s a vital part of personal, social, and even global harmony, not just something we engage in when it’s convenient. That is why, the mission of empowering action through climate justice education in settings that are not traditional elitist classrooms, but other ones that help democratize knowledge. Because it is believed that most humans do the best they can with the knowledge that they have, this page is on a mission to make that knowledge accessible, easier to understand, and empower others to use that knowledge as power. People traveled from nearly and faraway places to attend the conferences based on these matters. Participants learned about the importance of incorporating intersectional queer ecofeminism into climate disaster response management, and how, as individuals and as a community, they can reshape what happens during -what has unfortunately become our reality- climate change induced catastrophes. The engagement metrics of this page is 3.31% and the hashtags used are **#EnvironmentalJustice** **#Sustainability** **#Intersectionality** **#SocialJustice** **#InclusiveFuture** **.**

Another page which is examined was created on August 7th, 2020. The engagement metrics of this page is 1.18%. It is a pan Africa movement that brings off local climate measures such as the promotion of the reclamation of Lake Chad, raising consciousness about climate induced problems in disputed zones and African societies for demobilization, regional strength and the sustenance of livelihoods. This page tries to prove that peace is the ability to sustain one’s livelihood. The cause of action is to take proper measures to face climate change. The goal of this page includes: embracing for the restoration of Lake Chad, constructing a smart education for climate justice by involving youth, increasing solutions across sectors, advocating green democracy. The hashtags used in this page #climatechange #lagosnigeria #flood. The next Facebook page selected for the paper is created to discuss facts, information and issues about topics related to Feminism. The main aim of the page is to enlighten readers with books, links, documents and peer reviews shared by the members of the group. By sharing and reading about various topics on feminism, this will act as a support system and advice for women.

Moreover, there is another page that suggests the correlation of women and nature. In this page, the concept of Ecocide has been highlighted through different posts. Ecocide is defined as the most dreadful form of environmental demolition. According to Thomson Reuters Foundation, Ecocide is considered a crime in 11 countries including Russia and Ukraine.

YouTube stands as the most widely used video platform globally, with users consuming 4 billion hours of video monthly and uploading 72 hours of content every single minute. YouTube was founded by Chad Hurley, Steve Chen and Jawed Karim in the year February 2005, and named it “ YouTube.com”. People started to create video pages through the YouTube platforms on which users could upload, view, and share photos. Eventually, YouTube has gained a lot of users including scholars and educators.

Now, coming to the analysis of ecofeminism in YouTube channels, the first page which is selected is a page which is created to enlighten people about movements which are related to environmental problems. The page requests users to join the group and give new solutions to protect the earth and all lives on Earth. The engagement metrics of this page 0.0036% and the hashtag used in this page are #FarmerCase.

In this panel discussion, it is said that women in India are protectors of the environment. The next page which is selected mainly consists of videos related to burning planets. The disparaging difference lies in a more intense debate than fired and discharged landscapes. There is a distinction between burning living biomass and burning fossil biomass. Their occurrence is evident as they rage from the Arctic to the Amazon, and from New South Wales to the West Coast. They are conspicuous, and their smoke signifies their existence through the extensive smoke that drifts far away from the actual flames. The engagement metrics used in this page is 1.53% and the hashtags used in this page are #consumerism #shopping #climatechange.

In one of the environmental video essays related to consumerism, we study greenwashing using Fiji Water’s marketing campaign as a case study. We explore why green products are not necessarily as eco-friendly as their packaging products. It seems to be almost everywhere these days that if we walk into any grocery store, we see products related to green marketing mentioning that ‘our eggs are natural, and our shampoos have green labels with pictures of trees and leaves.’ In various respects, the movement towards eco-consciousness and responsible consumerism is a beneficial shift. To minimize both industrial and individual environmental impacts, organic foods and sustainably produced clothing are essential. However, concealed within these ethically sourced and environmentally friendly items is a deceptive form of marketing. Greenwashing is prevalent across nearly every industry in the consumer market, from personal care items to meat packaging. Today, let’s examine how Fiji water utilizes advertising campaigns to build an eco-friendly image for a company that harms the environment. Greenwashing is considered problematic because it capitalizes on consumers’ aspirations to lead environmentally conscious lives while not necessarily offering a sustainable product. On a more profound level, one of the most sustainable actions is to purchase fewer items. Therefore, regardless of the product’s quality, it can still be somewhat misleading to promote it as environmentally friendly. So greenwashing means to use titles that are all eco- friendly and natural. Moreover, using green labels in order to attract a customer to buy it is by no means environmentally friendly. At times, there are some companies that falsely label their products as eco-friendly like eggs that are labeled as farm fresh. Fiji water’s marketing campaign epitomizes the nature of this greenwashing. Fiji water has been portrayed to be environmentally friendly. But the biggest negative side of Fiji water is using plastic bottles shipped to destinations around the world. These practices create destruction in both the environment and the world. They are chasing green- minded consumers by formulating their water bottles as completely environment friendly products.

The next YouTube channel which has been taken for this paper is a pan Africa movement that performs grassroot based climate change such as endorsing the reclamation of Chad Lake, uplifting awareness about climate induced issues in disputed zones and African societies for demobilisation, regional solidity and the provision of livelihood. We assume that peace is not just the dearth of war, it is also the capacity to support one’s livelihood. The reason behind taking proper action is people can’t isolate or quarantine themselves in this climate change. The mission of this channel is to build a climate bright training through youth participation.

The next YouTube page selected for the paper is an Indian couple but lives in Australia *.* The page is about a couple who quit their engineering jobs and go out to live in a van to travel round the world. They make travel blogs about their daily life, share their experience of living in nature and travelling the whole world. They move from place to place through their van and explore the various food, scenery and culture of that place. The engagement metrics of this page is 0.271% and the hashtags used here are mainly #travel, #Hindi etc. The audience posts excellent comments based on their world trips.

There is a post taken in the Daintree rainforest, which is the world’s oldest rainforest in Australia. The Daintree rainforest is one of the oldest rainforests in the world. It is a 135 million years old rainforest located in Tropical North Queensland, Australia. It is over 1200 square kilometers and many plants and animal species are found in this area which are not found elsewhere.

Furthermore, there is another page is a feminist worldwide promotion organisation for the natural world, gender and environmental justice that nurtures and safeguards natural rights, gender equality and the coherence of the environment for the world.

Another post is about a conference named Convention on Biological Diversity (CBD) Women’s Caucus held on 15th October, 2012 during the 11th Conference of parties in Hyderabad. In this conference, Vandana Shiva attended this conference as a keynote speaker where she shared stories from her lifetime of protester and research on women, environment and social egalitarianism and motivated the room of next generation activists to ‘work actively’ to keep impelling for a new productive representation which earth centralised and women centralised.

On one hand, women are the ones who create every single popular post on Instagram blogs and on the other hand, there are also some posts on YouTube and Facebook where the theory of ecofeminism and its broader perspective is discussed and investigated in a group. Women who have encountered health issues, such as infertility or pregnancy complications are being discussed in some groups. It is crucial to explain the relationship between the toxins in the environment and women’s health. Some of them draw attention to the fact that they are mothers because they believe that it is essential for their children’s health to lead a natural, sustainable life devoid of excessive substances. The majority of the posts thus play on women’s natural tendency toward caution; several of the stories make a clear connection between women’s health and environmental deterioration. For instance, some discuss how the quality of the air, water, and cleaning products used in the home affect women’s health, including hormones, fatigue, and fertility; how the mineral cadmium, which is found in accessories for women, is injurious to both human health and the environment; and how hazardous petroleum is commonly found in clothing. In addition to discussing the detrimental effects of toxic chemicals on women and the environment, the majority of influencers discuss gendered sustainability issues, such as women’s sustainable clothes, as well as reusable period and cosmetics products.

Additionally, strong photographs accompanied by compelling statements of action are frequently used in Instagram and Facebook activism. This is referred to as “visual activism,” which is a term used to describe artistic movements and activism that employ performance and visual elements to further a cause ( San Cornelio et al., 2021). Environmental activists hope to evoke strong feelings and reactions in viewers and awaken their sense of duty to protect the environment by fusing compelling text with striking imagery. Furthermore, digital photos offer context and proof for real-world occurrences, which may make protests safer for women and other vulnerable groups ( Highfield and Leaver, 2016; Tuli and Danish, 2021). Strong language like “people not profit” and “no planet B” is used in the captions to emphasize how urgent the climate problem is and how important it is that more people understand how intertwined humans, the system, and climate change are.

Additionally, by gathering support to educate, identify, and inspire individuals to engage in activism, hashtags created to start environmental movements can also help bring about social change. As per Moscato (2016), hashtags can be employed in online activism to modify public opinion and elevate activism’s standing on social media inside the public domain, particularly concerning environmental matters. To get the attention of the public, it must be included in online activities and movements that have a strong message in the media ( Hassler et al., 2021). Furthermore, using the term “woman” or “female” prominently in their advocacy signifies that women are essential and at the center of activism. As a component of internet activism, the hashtag movement can increase social media involvement and even mobilization. Furthermore, social media is utilized to assist both current and historical activities in addition to showcasing the environmental movement that activists started. The use of certain hashtags pertaining to women and the environment serves as an example of this, emphasizing the necessity of positioning women as key players in achieving sustainability and finding solutions for environmental issues.

Thus, it is found that women are the knowledge producers in this illustration. Instagram, YouTube and Facebook provides users with access to their knowledge without casting doubt on their abilities. Their knowledge is valued based on their experiences as mothers and/or women, rather than being investigated and peer-reviewed as they would if they approached a scientific journal. From a feminist perspective, it is obvious that the social media community values the common knowledge that arises from working in the home and taking care of food, recycling, kids, and other responsibilities. This is also evident in the way the influencers discuss the importance of online community building to them.

In general, the influencers use their followers’ health and well-being as the primary driving force to encourage them to be more sustainable or to monitor their sustainable journeys. Their followers are primarily women. The environment’s health and well-being are closely linked to the health and well-being of individual women, and vice versa. Products that are bad for one are also considered bad for the other. The majority of the accounts seem to agree that the chemicals used in most products make modern life unhealthy. These are the household items that are used daily, such as household items, cooking equipment, kid toys and so forth. Nonetheless, women are taking it upon themselves to spread the word and criticize this way of life on Instagram, Youtube, Facebook. They can create a community of women on this platform who have similar life experiences and will be impacted by these toxins in similar ways because of their femininity. Nevertheless, some products are also marketed using the legitimacy that their female followers grant them.

Most of the women who manage these accounts rely on these brands as their main source of income, so gender sustainability is used to promote them. Since they identify as environmentalists, this does not necessarily invalidate their sustainability journey or motivation. The majority of the sustainability influencers in this study talk about “slowing down” and “natural living,” which challenges the capitalistic way of life that promotes perpetual growth. One influencer’s comment alludes to a desire to return to life as it was before our modern lifestyles. Ecofeminism can be seen in this rejection of neoliberal values and in the emphasis placed on empathy and a connection to the natural world.

The majority of female users discuss the value of the platform’s community, and the fact that women make up the majority of their followers indicates that women use these social media platforms to share their knowledge about sustainability with other women, who then validate it. This knowledge is based on anecdotal and personal experience rather than scientific fact. As a woman or mother, you are typically the primary caregiver for your children and the one who handles housework. This is explained by feminist perspective theory, which holds that women’s experiences of marginalization in a world ruled by men have given them important knowledge. These accounts convey a powerful message about the female community’s support of one another; the majority of these accounts like and comment on each other’s posts and identify these communities as the main reason they use the platform. Consequently, Instagram, YouTube, Facebook serves as a platform for women who are burdened with housework and the associated emotional anxiety to share this burden with other like-minded women. This is because, as ecofeminism makes clear, taking care of the environment is viewed as a value that is primarily held by women. As a result, these influencers feel that they must make their homes more environmentally friendly, with their partners simply choosing to “hop on board” or “tag-along”, as some of the Instagram influencers have put it.

## Conclusion

The exploration of media within the realm of ecofeminism has illuminated both the successes and challenges in representing the interconnected struggles of gender and the environment. Instagram, YouTube, and Facebook allow people to share what they know without questioning their skills. The experiences of mothers or women are what give their knowledge value, unlike the rigorous checks that come with a scientific publication. Looking at it from a feminist angle, it’s clear that social media appreciates the everyday wisdom that comes from managing a home, preparing meals, recycling, caring for children, and handling various responsibilities. Through content analysis and case studies, this research has provided valuable insights into the ways ecofeminist themes are portrayed, disseminated, and engaged across diverse media forms. This research not only sheds light on the current state of ecofeminist representation in media but also issues a call to action. Media creators, educators, and activists are invited to leverage the findings to strengthen the ecofeminist movement. This research serves as a stepping stone toward a more robust, inclusive, and impactful ecofeminist discourse. By critically examining the representation of ecofeminist themes in media, we contribute to the ongoing dialogue surrounding environmental sustainability, gender equity, and the vital intersection between the two.

## Ethics and consent

Ethical approval and consent were not required.

## Data Availability

The data used in this study has been collected from publicly available Instagram, Facebook, and YouTube pages for the purpose of analyzing themes related to women and nature. We have collected the data from these social media accounts based on themes, hashtag used, engagement metrics and audience interaction etc. Due to ethical considerations, including the lack of consent from the original content creators, the data has been de-identified to ensure that no personally identifiable information is present.
