# Women and nature: Ecofeminist study in social media

**DOI:** 10.12688/f1000research.162436.2

**Published:** 2025-07-16

**Authors:** Jhilik Chakraborty, Arpita Goswami

**Affiliations:** 1 Department of Humanities, School of Liberal Studies, Kalinga Institute of Industrial Technology (KIIT) Deemed to be University, Bhubaneswar, Odisha, 751024, India; 2 Department of Humanities, School of Liberal Studies, Kalinga Institute of Industrial Technology (KIIT) Deemed to be University, Bhubaneswar, Odisha, 751024, India

**Keywords:** Ecofeminism, Women, Nature, Digital Platform, Social media, Facebook, Instagram, Youtube, Representation

## Abstract

**Background:**

This research examines how ecofeminist themes are presented and shared on digital platforms. It focuses on three widely used social media networks, namely YouTube, Instagram, and Facebook to explore how their features, such as videos, reels, posts, and images, convey ecofeminist ideas and principles in online spaces.

**Methods:**

We have adopted a purposive, qualitative, and thematic analysis method. Data have been manually collected from the selected social media platforms using keywords like “ecofeminism” and “women and nature.” Content has been selected based on thematic relevance. Only publicly available materials have been used.

**Results:**

Instagram, YouTube, and Facebook each contributes uniquely to the ecofeminist discourse by offering visually engaging reels and images, academic and artistic content, and select group and page-based advocacy, respectively. Through various digital entities such as groups, pages, reels, videos, and pictures, many social media accounts vividly demonstrate the close relationship between women and nature, the exploitation they face from patriarchal society and how they can be protected. Despite this vivid portrayal, the content across platforms remains scattered and inconsistent.

**Conclusion:**

The study highlights both the potential and the limitations of social media in presenting ecofeminist narrative. It emphasizes the need for consistent and dedicated efforts to promote ecofeminist values online. The study adds to the fields of ecofeminism and media studies by showing how online content can reflect, support, and shape ecofeminist thinking today.

## Introduction

In contemporary society, both the environment and women continue to face pressing challenges ranging from environmental degradation to systemic gender-based exploitation. In a country like India, women are often revered and symbolically elevated to the status of goddesses. However, despite this cultural idealization, the lived realities of many women sharply contradict these traditional values. On a daily basis, women encounter various forms of violence, including domestic abuse, sexual harassment, marital rape, and dowry-related offenses. Alongside physical violence, widespread gender discrimination persists in many spheres of life. Women frequently receive lower wages for equal work, face barriers to leadership positions in politics and business, and experience significantly higher rates of domestic and structural oppression. Simultaneously, the environment is being severely impacted by factors such as rapid population growth, urbanization, overconsumption of energy, and unsustainable modes of transportation. These developments have accelerated deforestation, pollution, and resource depletion, contributing to a broader ecological crisis. In response, several theoretical frameworks have emerged to address these dual forms of exploitation such as environmentalism, feminism, ecocriticism, and ecofeminism etc. Environmentalism, which gained momentum in the early 19th century, advocates for the protection and preservation of the natural world. It encourages behavioral change at both individual and systemic levels to reduce environmental harm and promote sustainable living. The movement also calls for political, economic, and social systems that uphold ecological responsibility and foster a more balanced relationship with nature. Feminism, on the other hand, seeks to dismantle structures of gender inequality and promote the rights and autonomy of all genders. Feminist thought challenges pay disparities, restrictive gender roles, unequal access to healthcare and education, and other manifestations of patriarchal dominance.

Ecofeminism merges the core principles of both feminism and environmentalism. It posits that the oppression of women and the exploitation of nature are deeply interconnected, both rooted in patriarchal and capitalist systems. While feminists analyse the causes and consequences of gender-based oppression and environmentalists focus on the causes of ecological degradation, ecofeminists identify a shared logic of domination—particularly a patriarchal worldview—as the root cause of both. According to Sturgeon (1997), ecofeminism represents “a double political intervention, of environmentalism into feminism and feminism into environmentalism” (p. 169). Françoise d’Eaubonne first coined the term “ecofeminism” in her 1974 work Le féminisme ou la mort, and since then, the term has evolved into a theory focused not only on critique but also on practical transformation, often referred to as “quilt theory” ( Patil, 2020). Prominent scholars such as Vandana Shiva, Bina Agarwal, Carol J. Adams, Ynestra King, Maria Mies, and Greta Gaard have emphasized the interconnectedness of various forms of oppression, including the domination of women, nature, and animals within patriarchal systems. Influential works such as Staying Alive: Women, Ecology, and Development ( Shiva, 1988), Ecofeminism ( Mies and Shiva, 1993), and The Sexual Politics of Meat ( Adams, 1990) have laid the groundwork for ecofeminist critique and activism. According to Gaard (2015), patriarchal attitudes are the common root behind both environmental destruction and the exploitation of women. Patil (2020) outlines three core tenets of ecofeminism that guide our study: (1) the connection between women and nature, often rooted in women’s dependence on and care for natural resources; (2) the nurturing role of women as environmental protectors; and (3) the recognition that patriarchal development degrades both women and nature. As such, ecofeminism not only critiques these systems of domination but also advocates for inclusive, equitable, and sustainable relationships between humans and the environment. It calls for dismantling the power structures that enable exploitation and promoting ethics of care, interdependence, and justice. Although originally grounded in academic discourse, ecofeminism has increasingly moved beyond scholarly spaces. Its principles are now being actively practiced and promoted through digital media platforms.

In today’s media-saturated world, social media has become an essential part of everyday life, especially platforms like YouTube, Instagram, and Facebook, which are widely used across demographics for entertainment, communication, and commerce. These platforms have evolved into powerful tools for advocacy, enabling content creators, educators, activists, and community organizers to reach large and varied audiences. Through images, short-form reels, long-form documentaries, personal reflections, and educational tutorials, creators are actively spreading ecofeminist messages. This includes not only environmental concerns but also the intersectional links between gender, ecology, justice, and care. From grassroots storytelling to academic dissemination, social media has amplified ecofeminism’s reach, bridging the gap between scholarly theory and lived experience. As a result, platforms that were once seen primarily as entertainment spaces are now central to the global conversation on ecological and gender justice. Several academic works have explored the intersections of feminism and digital media, offering insights into how feminist discourse is articulated and expanded through online platforms. For example, Garcia-Ruiz et al. (2021) examine feminist posts on a Facebook page that focus on topics such as the body and sexuality which are historically shaped by oppressive language and legislation. Their study contributes to an understanding of how digital spaces create new opportunities for women in Latin America to express personal and collective agency while resisting patriarchal narratives. Similarly, Pruchniewska (2019) investigates how private Facebook groups function as modern-day equivalents to second-wave consciousness-raising groups. Through interviews with 26 women, she reveals how these Facebook groups serve as safe spaces where women discuss professional experiences and share advice. These conversations often extend beyond the digital realm, influencing women’s lives offline and reinforcing feminist values. On YouTube, the feminist discourse has also gained momentum. Serrano-Contreras (2021) explores the evolution of feminism on YouTube, particularly in the Spanish context. Using Natural Language Processing (NLP), the study identifies a rise in conversations about Spanish feminism post-2016, reflecting a growing public engagement with feminist ideas. However, in contrast to the growing body of literature on feminism in digital spaces, there is a lack of works focusing on the promotion of ecofeminism through digital platforms. Nevertheless, a few notable works address this gap. For instance, Singh and Bali (2022) examine the dynamic relationship between media, literature, and ecofeminism. Their work explores how digital media—particularly Instagram—shapes and disseminates ecofeminist narratives, contributing to a deeper understanding of how visual platforms can reinforce environmental and gender justice. Jain (2020) focuses on the impact of digitization on women’s movements, especially in developing countries like India. By employing postcolonial and postmodern feminist frameworks, Jain assesses the benefits and limitations of digital activism. The study highlights that while online spaces can replicate the hierarchical structures of global political economies, they also offer new avenues for feminist solidarity and ecofeminist engagement. Jain further argues that feminist movements must remain aware of cultural contexts in digital advocacy and calls for the creation of laws and public policies to address online misogyny, viewing it as a structural problem rooted in longstanding gender inequalities.

To the best of our knowledge, we have not encountered any existing academic works that specifically examines the combined role of YouTube, Facebook, and Instagram in promoting ecofeminism. In this paper, we aim to fill that gap by exploring how these three platforms collectively contribute to the dissemination and advocacy of ecofeminist ideas and themes.

## Methods

In this study, first we have selected three social media platforms, namely YouTube, Instagram, and Facebook for collecting entities such as groups, pages, accounts, channels, images, short videos and long videos related to ecofeminist themes. These three platforms have been selected because they are among the most widely used social media networks globally, including in India, where both authors of the current paper are based. These platforms are used extensively by people from diverse social, cultural, and professional backgrounds and offer a wide range of personal, professional, entertainment and educational content. The data collection and analysis have been conducted manually.

We have searched data in Instagram by using keywords “ecofeminism”, “ecofeminists” and “women and nature”. First, we have visited “Account” section where we have found several public and private profiles, some in English and some in other languages. Many older accounts are inactive, while some newer ones have limited engagement, typically under 500 followers with few likes, comments, or shares. Among public English-language accounts, most of the content is loosely related to ecofeminism. We have not found any account consistently focusing on ecofeminist discourse. Therefore, we have moved to the “For You” tab, where we have found diverse collection of reels and images relevant to ecofeminist themes uploaded from random accounts which are not directly related to ecofeminist themes. From this section, we have selected reels based on both view count and thematic relevance, and images based solely on thematic relevance. The sample included 4 reels with over 100,000 views, 7 reels with 20,000–100,000 views, 6 reels with fewer than 1,000 views, and 10 ecofeminist-themed images. Relevance and thematic clarity have been prioritized over metrics like likes or comments. Only content from the “For You” section has been used for analysis.

For YouTube, we have used the keywords “ecofeminism”, “ecofeminists” and “women and nature” and applied the filter ‘relevance’. We have not found a single channel which is completely dedicated to ecofeminist themes. However, there are a lot of videos on ecofeminist themes. Only long English videos related to ecofeminist ideas and principles have been selected for analysis. We have categorized the data into two segments. First, we have selected popular educational videos on ecofeminist themes uploaded between 6 and 14 years ago with viewership ranging from 9,000 to 110,000 views. These include academic lectures, talks, expert interviews, and advocacy material. The second category videos are recent and diverse content videos uploaded within the last five years. These recent uploads encompass poetic documentaries, artistic reflections, workshops, and poetry on ecofeminism.

For Facebook data, we have searched using the same keywords such as, “ecofeminism”, ecofeminists” and “women and nature” in the search bar. First, we have clicked on the “Group” section. We have found very few public groups on ecofeminist themes. The groups that exist are generally less active, and the number of posts is very limited. Some groups have a good number of members, while others have fewer. Even though the group names refer to ecofeminism, the content in most of them is not entirely related to ecofeminist themes. Considering all these observations, we have selected three English-language groups with both high and low member counts that contain some direct posts related to ecofeminist principles and ideas. Then we have explored the “Page” section. Similar to the groups, the number of pages focused on authentic ecofeminist themes is quite limited. However, the existing pages are relatively more active in terms of content uploads and engagement. We have selected four such pages based on a combination of high likes, followers, and content relevance. For analysis, we have selected specific posts from these groups and pages that are directly aligned with ecofeminist values and principles. We have also explored reels uploaded on Facebook related to ecofeminist themes. However, it is found that the number of such reels is very limited. Moreover, the few reels that have gained a significant number of views on Facebook are also available on Instagram. Therefore, our discussion of these reels has been included in the Instagram analysis. Based on the discussion, it can be concluded that our data collection method is purposive, qualitative, and thematic in nature.

Regarding the time period of data collection for the paper, it is mentioned that the data has been collected in two phases. The initial phase has been taken place from December 2024 to April 2025, marking the beginning of our work on this study. Subsequently, the second phase, from May to June 2025, has been carried to incorporate additional content. With respect to the date of content creation, It is revealed that none of the selected content was created before 2010. The data created between 2010 and June 2025 have been selected for the study. Its also mentioned that only publicly accessible content from social media platforms have been used in this study. We have not sought individual consent from content creators. However, to ensure ethical integrity, we have de-identified all examples used in the analysis.

As far as data analysis is concerned, this study has employed a manual qualitative content analysis approach. No software tools or automated coding schemes were used. We have closely examined selected digital entities such as pages, groups, accounts, images, and videos from YouTube, Instagram, and Facebook. Their content has been explored in detail to analyze how captions, visuals, symbolic elements and language conveyed ecofeminist ideas and themes. This included identifying representations of the woman and nature relationship, critiques of exploitation of both, and calls for protection and preservation. Since the objective of analyzing each digital entity is the same, we have applied a uniform analytical approach to both the selection and analysis of all entities. Both authors have carefully reviewed each piece of content. They have discussed and mutually agreed on its thematic relevance and meaning to ensure consistency and reliability. The flowchart illustrating the method is provided below:

**Figure 1.**
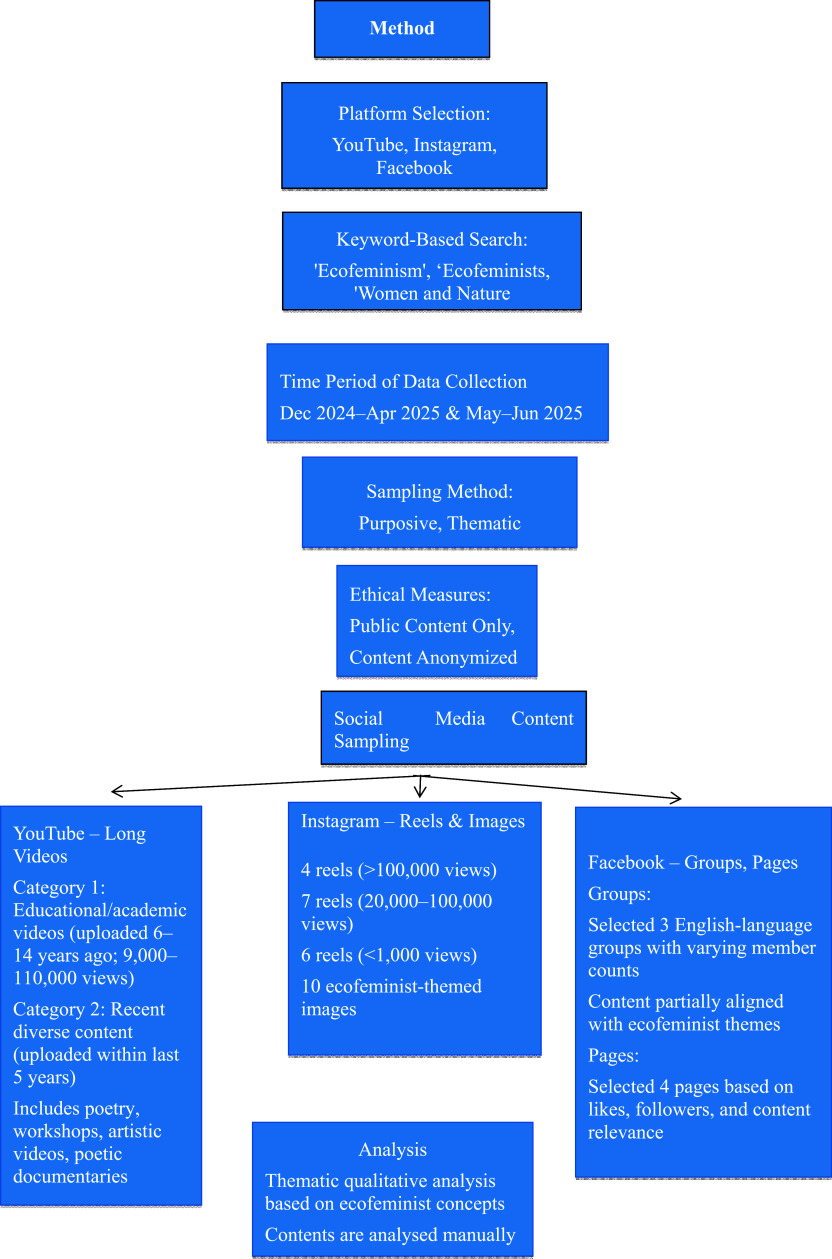
Flowchart of data collection and analysis method.

## Results and Discussion

Ecofeminism, as a recognized literary theory, is included in the syllabus of various literature, gender studies, and environmental humanities courses. YouTube plays a significant role in the dissemination of academic tutorials and educational content related to such subjects. Consequently, most of the videos related to ecofeminism uploaded on YouTube consist of tutorials and lectures intended for academic and instructional purposes. Several of these videos explain core concepts of ecofeminism, such as the roots of ecological feminism, value dualism, the ethics of care, and the parallel domination of women and nature.

For instance, one tutorial (42,413 views) outlines key theoretical discussions and foundational concepts. Similarly, another video (9,481 views) addresses ecofeminism within Indian literary contexts, offering definitions and examples from regional texts. Another tutorial (9,000 views) provides conceptual discussion, while a separate video (1,455 views) delivers a concise explanation of the relationship between ecology and gender. Moreover, some educational videos incorporate multimedia elements to enhance engagement. For example, one video (8,810 views) narrates the concept of ecofeminism while showing images of prominent ecofeminist thinkers and clips of women interacting with nature. Another popular video (46,402 views) combines animation with climate education to introduce young people to ecofeminist activism. These presentations not only inform viewers but also foster emotional engagement and accessibility.

In addition, some other videos that blend academic insights with artistic expressions to convey ecofeminist ideas include one (341 views) that examines ecofeminist art and activism through a researcher’s lens, another (204 views) revisiting ethical theories addressing themes such as value dualism and the ethics of care, and a series (586 views across 26 videos) offering lectures, interviews, and workshops that center spirituality, ecology, and decolonial perspectives. Apart from them, several other videos present experiential content, highlighting personal narratives that humanize ecofeminist themes. For example, one talk (51,000 views) features a speaker who shares her family’s struggle with cancer and infertility, linking personal trauma to chemical exposure and environmental degradation. Drawing on the works of ecofeminist thinkers, she describes how the ethics of care central to ecofeminism helped her find strength and meaning through teaching and activism. Similarly, another talk (29,173 views) addresses gendered exposure to toxins, breast cancer risks from parabens in beauty products, and maternal health.

Furthermore, more recent content expands ecofeminist discourse to include themes such as queer theory, veganism, and indigenous activism. For example, one video (4,025 views) features a dialogue between two academics who discuss dismantling gender and species binaries. Meanwhile, another video (578 views) presents a lecture exploring how food politics, animal ethics, and feminist theory intersect. The speaker critiques how toxic masculinity shapes environmental behavior and argues that the human-animal divide perpetuates broader systems of violence. These discussions signal a shift toward more intersectional understandings of ecofeminism. Additional content revisits grassroots struggles led by rural and tribal women in India, linking ecofeminism to anti-colonial land rights activism. In parallel, several videos spotlight foundational thinkers. One prominent figure appears in multiple interviews (68,889 views; 75,055 views; 28,702 views) discussing globalization, spiritual ecology, and indigenous knowledge systems. Another influential voice is featured in a video (7,702 views), elaborating on integrative activism and post-development theory. Meanwhile, a personal reflection video (7,274 views) offers insights into engaging with ecofeminist ideas.

Alongside these accounts, other creators turn to artistic and creative forms to articulate ecofeminist messages. A particularly poignant example (1,268 views) adopts a lyrical and emotive tone to articulate the themes of ecofeminism. As the visuals move through forests, rivers, and maternal imagery, a female voice narrates the intimate bond between women and nature. “Mother Nature gives us life, heat, food, pure air to breathe, water to drink, and land to stay,” the narrator explains, emphasizing that nature is a source of nourishment and protection. The documentary parallels these life-giving forces with the unique role of women, particularly mothers, as caregivers and preservers of the human race. It highlights the sacrifices women make and their nurturing role in raising children, reinforcing the idea that both women and nature sustain life yet are often undervalued and exploited. Scenes of environmental destruction, land strewn with garbage, deforestation, and air pollution are juxtaposed with social neglect and violence against women, drawing a powerful analogy. The documentary concludes with an urgent call to action, just as we must protect and respect our environment, we must uplift, educate, and safeguard the rights and dignity of women. It warns that humanity itself cannot survive without women and nature.

Another poetic documentary (631 views) explores the beautiful relationship between women and nature. It draws parallels between the rhythms of a woman’s life marked by change, crisis, and joy and the cycles of nature, such as seasonal flourishing and natural calamities, suggesting a deep connection between the two. Likewise, another video (1,046 views) blends music and storytelling to interrogate disconnection from nature in urban settings. A separate artistic video (384 views) explores the relationship between ballet and ecological thought, showing how embodied art forms can convey environmental resistance and philosophical reflection. One poetry video (61 views) blends ecofeminist critique with radical political commentary. The poem criticises systemic structures like patriarchy, capitalism, colonialism, and ableism, exposing how these forces marginalize vulnerable groups, silence voices, and destroy collective support systems. It offers a fierce resistance to regressive ideologies while emphasizing the potential of intersectional ecofeminism approach. Another poetic video (56 views) features a powerful visual metaphor like a green field shaped like a reclining woman, her arms outstretched, hair flowing, and closed eyes. Animals, rivers, and people appear across her body, symbolizing the interdependence between nature and life. The poem praises her as the source of nourishment, whose breasts are like hills, whose tears form rivers, and whose strength is supernatural. Rooted in African imagery and Ubuntu philosophy, the poem highlights both her life-giving power and the violence of exploitation, referencing mining in Namibia and the spiritual wisdom of women rooted like ancient baobab trees.

A video essay (428 views) critically interprets a mainstream film through an ecofeminist lens. The speaker first contextualizes climate change discourse before exploring the film’s narrative, in which a woman lives peacefully with her husband in a remote home until a series of disruptive guests enter their sanctuary. The woman, a homemaker, is portrayed as a symbolic embodiment of Mother Earth, nurturing and generous while the poet-husband and intrusive guests serve as metaphors for patriarchal and anthropocentric exploitation. The anchor argues that the film’s chaotic scene mirrors environmental collapse and the silencing of feminine care and intuition. Equally important are those videos that highlight the internal tensions within the ecofeminist movement. For instance, one popular video (117,853 views) opens with striking imagery of environmental disasters and draws on foundational texts, while simultaneously questioning the movement’s historical limitations. It critiques the dominance of white Western perspectives and calls for more inclusive approaches that integrate race, class, and disability concerns. These thoughts are echoed in another panel discussion video (4,096 views) on the contemporary status of ecofeminist politics.

Moving into Instagram, we have observed a wide range of reels and images related to ecofeminist themes uploaded by various accounts. Among the most widely viewed content on Instagram is a reel with 749,000 views which tells the inspiring story of Piplantri village in Udaipur, where a tradition inspired by ecofeminist principles mandates planting eleven trees for every girl born. This initiative was taken by Sarpanch Shri Shyam Sunder Paliwal following severe drought and personal loss. He transformed 9,000 acres into greenery and symbolically links women’s births with ecological restoration. The reel has garnered 70,600 likes and 436 comments. Similarly, a very impactful reel (348,000 views) addresses an issue faced by women agricultural labourers in Maharashtra. The reel reveals how women labourers have been forced to undergo hysterectomies because climate change has increased the demand for physical labour, especially in harsh conditions. These surgeries are done to prevent menstruation and pregnancy so that women can work longer hours without interruption. The short video highlights the intersection of environmental degradation, gender oppression, and exploitative labor practices. Through an ecofeminist lens, this situation shows how patriarchal and capitalist systems treat both women’s bodies and nature as things to be used and thrown away. Women and nature are valued only for what they can provide, not for their well-being and rights. By using hashtags #ClimateChange, #WomensRights, and #EcoFeminism, the reel reaches audiences interested in both climate issues and women’s rights, showing how these topics are linked. The video has received 16,400 likes and 189 comments, reflecting growing public concern with the disturbing reality depicted in the video, where climate change and gendered exploitation intersect in the lived experiences of women labourers. In another reel (238,000 views and 14,500 likes), Vandana Shiva critiques patriarchal foundations in Western science and governance and emphasizes the role of ecofeminists in decolonizing knowledge and challenging hierarchies that subjugate women, nature, and marginalized communities. Many hashtags are used in this reel—such as #feminism, #indianfeminism, #indianfeminist, #desifeminism, #desifeminist, #southasianfeminism, #ecofeminism, #ecofeminist, #indianecofeminism, #desiecofeminism, #vandanashiva, and #environment—which help increase the visibility of the reel. Another popular reel (105,000 views) focuses on the history and meaning of the term “tree hugger.” where Vandana Shiva explains how women’s deep connection to nature and traditional knowledge have played a vital role in protecting the environment. The reel highlights two major moments in history. First, it tells the story of the Bishnoi community in Rajasthan, India, where in 1730, a woman named Amrita Devi and over 360 villagers gave their lives to protect trees from being cut down. They hugged the trees and declared, “A chopped head is cheaper than a felled tree.” Second, the reel shows the Chipko Movement of the 1970s in the Himalayan region, where women hugged trees to stop deforestation and protect their sources of food, water, and fuel. Today, the term “tree hugger” is sometimes used as a joke, but this reel reminds viewers of its powerful origin as a symbol of courage and resistance. The post received 100 likes and 126 comments, helping spread awareness about women-led environmental movements and ecofeminism.

Another reel (30,000 views and 16,100 likes) presents ecofeminism as a critical feminist philosophy linking the oppression of women to the exploitation of nature. It highlights both the strengths and criticisms of ecofeminist ideas. It challenges the notion that all women have an inherent connection to nature and emphasizing the importance of more inclusive, intersectional approaches. Bringing in cultural heritage, a reel explores the traditional Indian textile art form Kalamkari within ecofeminist contexts (29,500 views and 1,775 likes). By highlighting Kalamkari’s sustainable use of natural dyes and motifs inspired by plants, animals, and female deities, the reel connects the art form to feminine values and a deep respect for nature through eco-conscious practices. Furthermore, it connects this heritage to the Chipko Movement, a grassroots environmental protest led predominantly by rural women in the 1970s, which symbolizes ecofeminist resistance against patriarchal ecological harm. This reel uses hashtags like #LoveYourNature and #SustainableLiving to raise awareness about cultural traditions and promote ecofeminist activism through social media. Another reel (28,900 views and 1,502 likes) captures attention by asking a poetic question: “Can trees speak?” It contrasts human and natural perceptions of time. While humans tend to view time as linear, moving from past to present to future, trees experience time as cyclical, reflected in the rings within their trunks. The reel suggests that for trees, the past and future are held within the present, making them silent witnesses to history and symbols of continuity. It invites viewers to slow down and truly listen when they pass a tree, as trees may hold and whisper stories of the land, people, and time itself. The reel connects with ecofeminist thought by treating trees as living beings with wisdom, deserving of care, attention, and respect, challenging dominant views of nature as mute or passive. Unique hashtags such as @theislandofmissingtrees, @ecofeminism, @treehuggers, and @therearerriversinthesky enhance the reel’s visibility and thematic depth. A creative reel (24,700 views and 1,696 likes) shows a young woman reading a book in a snow-covered forest. This visual reflects women’s interest in creativity, storytelling, and self-expression. It also symbolizes the emotional connection many women feel with nature and literature. The serene and solitary setting highlights how reading can be a source of comfort, imagination, and inner strength for women. Through hashtags like @fairytaleaesthetic, @calledtocreate, @ecofeminism, @storymedicine, @romanticacademia, and @forestcore, the creator reaches out to a community that values creativity and nature. Another reel demonstrates the distinction between cultural and radical ecofeminism (23,200 views). It highlights that cultural ecofeminism emphasizes on women and nature connections and goddess worship, whereas radical ecofeminism focuses on dismantling patriarchal gender roles. It also points out that to make ecofeminism truly inclusive and intersectional, it is important to consider how race, economic status, and gender identity shape people’s experiences. In another activist-oriented reel by a climate activist (14,300 views), draws parallels between environmental destruction and the historical oppression of women. The speaker challenges patriarchal ideas about gender and nature and urges for major changes that include the voices of marginalized groups, especially Indigenous and Black communities. Hashtags like #ClimateJustice and #TakeAction highlight its strong activist message. Finally, a reel focusing on the intersection between climate justice and racial justice in the Democratic Republic of Congo (17,500 views) highlights the exploitation of cobalt miners, mostly marginalized artisanal workers within the green economy. It underlines the contradiction between environmental sustainability and ongoing racialized labor abuses. This post challenges viewers to critically assess the global inequalities embedded in environmental solutions.

An Instagram reel with the slogan “Empowered Women, Empowered Planet!” (606 views) highlights the connection between women’s empowerment and environmental justice. It reflects an ecofeminist perspective, which sees both women and nature as being affected by the same systems of exploitation, such as patriarchy and capitalism. The message is that when women are empowered, especially in leadership and decision-making roles related to the environment, communities become stronger in protecting natural resources, adapting to climate change, and promoting sustainability. The slogan encourages collective action, showing that gender equality and environmental care go hand in hand. Hashtags like #Ecofeminism and #ClimateJustice help the post reach wider audiences interested in these topics and promote ongoing discussions around feminist environmental action. A thought-provoking Instagram reel with 542 views highlights the deep connection between women’s well-being and the health of the planet. Using an ecofeminist perspective, the post draws attention to how patriarchal systems tend to exploit both women and nature. It suggests that women’s health, life expectancy, and overall well-being are directly linked to the conservation of biodiversity and environmental care. The reel emphasizes that a healthy planet is essential for the well-being of women everywhere. When women and nature are treated with respect and care, societies become more balanced, and capable of thriving. This post received 21 likes and included the hashtag #WomensMonth, helping it reach people interested in gender and environmental issues. A reel featuring ecofeminist scholar Vandana Shiva’s visit to a tribal village in Odisha on Women’s Day with 334 views highlights her powerful message about the importance of the feminine principle in creating a sustainable future. Shiva emphasises that the fundamental energy of the universe *Shakti* represents nature’s creative force and is deeply connected to women. She critiques capitalist patriarchy for treating nature as lifeless and instead celebrates women and nature as dynamic, powerful forces of creativity and renewal. Shiva warns that the ongoing climate crisis, species extinction, and environmental destruction signal an unsustainable future. In contrast, she calls for a paradigm shift that recognises the rights of Mother Earth, women, and all living beings. This vision, she insists, is not only necessary but unstoppable. In another creative reel with 111, pink is used symbolically to represent International Women’s Day. The reel explores how women can channel their creative voices to reshape cultural narratives, protect the environment, and build a more hopeful future. Artistic expression becomes a powerful tool connecting women with their inner strength, forging emotional and social bonds, and strengthening their relationship with nature. The reel suggests that through creativity, women can challenge established norms, and promote social and ecological justice. Art becomes both a mirror and a catalyst. It reflects women’s dreams and struggles while inspiring others to act. This collective creative energy builds resilience, nurtures community, and motivates change. By embracing creativity, women take on leadership roles in shaping a more equitable and sustainable world. The hashtags used in this post #Ecofeminism, #FemaleFrequency, #WomenInArt, #SustainableArt, #CreativeRevolution, and #FeministArt help connect the reel to global conversations on feminism, environmental justice, and artistic activism. A reel with 427 views pays tribute to the pioneers of ecofeminism in celebration of International Women’s Month. It features images of influential women who have shaped and advanced the ecofeminist movement. These figures have played a vital role in revealing the deep connections between environmental harm and social injustice, particularly focusing on how both women and nature are exploited under patriarchal and capitalist systems. The reel educates viewers about the origins and principles of ecofeminism, while also inspiring future generations to carry the movement forward. The reel includes visionaries such as Françoise d’Eaubonne who coined the term ecofeminism and other leading scholars and activists like Maria Mies, Sallie McFague, Rosemary Radford Ruether, Carolyn Merchant, Val Plumwood, Wangari Maathai, Susan Griffin, Karen Warren, Judi Bari, and Greta Gaard. The reel uses hashtags such as #Ecofeminism, #ClimateJustice, and #PlanetProtectors to increase its visibility and link it to wider digital conversations. Another reel (113 views) opens with a powerful visual. A woman is shown sitting and drawing a flourishing tree filled with flowers, fruits, and leaves. In the drawing, she depicts women are standing very close to the tree. This image serves as a symbol of deep connection between women and nature. As the visual unfolds, the speaker, an advocate of sustainable practices discusses ecofeminism and the disproportionate impact of the climate crisis on women across the globe. She introduces ecofeminism as a theoretical framework that connects the exploitation of women with the degradation of the environment. The speaker highlights how, in modern society, the idea of “human” is often aligned with masculinity, while “nature” is feminized. Both are objectified and valued primarily for their extractive potential, leading to environmental destruction and social inequality. The reel also emphasizes women’s long-standing role in ecological knowledge and activism. It refers landmark movements such as the Chipko Movement of the 1970s. Framed within the context of International Women’s Day, the reel includes hashtags such as #sustainablefashion, #sustainability, #womensday, #internationalwomensday, #consciousshopping, and #consciousconsumer.

Now we have discussed images shared on Instagram that explore ecofeminist themes. The most analytically rich image is a historic photograph from the 1970s Chipko Movement, where rural women are seen hugging trees to stop deforestation. These visuals capture the intimate bond between women and nature, portraying women as both nurturers and protectors of the environment. The inclusion of the historic photo emphasizes how women have resisted ecological destruction. This image reflects the ecofeminist idea that women are deeply connected to the environment because of their embodied roles in sustaining life, community, and natural resources. The act of hugging trees becomes a strong symbol of protest against patriarchal systems that exploit nature, turning everyday care into resistance.

As part of an educational initiative, a five-image carousel shared by a climate-conscious digital magazine presents a thoughtful introduction to ecofeminism. The first image features a girl standing in a jungle, using her hands as binoculars, with the word “ecofeminism” and question marks beside her—inviting viewers to explore and question the concept. The following slides in the carousel include clear definitions, historical context, and impactful statistics. One slide quotes a well-known ecofeminist thinker who said, “A sustainable society would need to incorporate the hidden work, interests, and experience of women.” The post explains that ecofeminism emerged during the 1970s–80s and includes a statistic noting that women are significantly more vulnerable to climate-related disasters. Hashtags such as #ClimateChange and #GenderEquality helped extend the post’s educational impact. Another notable image focuses on Earth Day. It links feminist and environmental movements through the lens of ecofeminist justice. It explains how systems like patriarchy, colonialism, and capitalism oppress both the environment and marginalized groups, especially women, girls, and gender-diverse people. It calls for a shift from exploitation to cooperation, and from hierarchy to sustainability. By highlighting the leadership of marginalized voices who are often excluded from decision making yet central to environmental action, it reinforces the call for inclusive justice. With hashtags like #EarthDay, #FeministJustice, and #EnvironmentalJustice, the image offers a meaningful voice in the ecofeminist digital space. Following this, another image features a woman gently cradling a tree growing from a globe, symbolizing the Earth. Her calm expression and the tender way she holds the globe convey a nurturing bond between women and nature. The poetic caption emphasizes ecofeminist themes, portraying the woman not just as a guardian of trees, but as a protector of all life. Hashtags like #Ecofeminism, #WomenForNature, and #WomenForLife strengthen this message and connect the image to broader activist and digital communities. The background audio, taken from the movie Moana, celebrates feminine strength and ecological harmony. In continuation, an image highlights ecofeminism by focusing on the resistance led by Waorani women in Ecuador’s Amazon rainforest. It shows how these Indigenous women are defending their land and environment from exploitation. Through their activism, the image illustrates how ecofeminism and Indigenous resistance are deeply connected, both rooted in care for the Earth and the rights of marginalized communities. Their defense of ancestral land is not just a protest against oil extraction, it also stands against the way powerful institutions like the state, corporations, and patriarchal systems try to take away women’s power and voice. The Waorani women see the Earth as a living being and believe in a relationship of mutual respect and care with nature. Their activism draws on the knowledge passed down through generations and their close, everyday understanding of the environment. Hashtags such as #Ecofeminism, #IndigenousMonth, and #ClimateAction help spread the message to wider audiences, while additional hashtags like #MujerForestal, #TerritoryAndResistance, and #FeministReads encourage exploration through activism and literature. Building on these layered perspectives, another image makes a connection between ecofeminism and Sankhya Philosophy. Sankhya posits two foundational principles, namely Purusha (consciousness) and Prakriti (nature). Prakriti, associated with feminine energy, resonates with ecofeminism’s celebration of the feminine and natural world. Sankhya’s holistic view of the universe reflects ecofeminism’s emphasis on interdependence, the blurring of binaries, and critique of patriarchal systems that exploit both women and nature. This image with hashtags like #Ecofeminism and #Sankhyadarshan offers an important cross-cultural philosophical perspective. Another post consists of nine image slides that tell an emotional story about our connection with the Earth. Each slide has simple drawings and powerful messages. The post says that the Earth remembers pain, damage, and also care. It highlights the message that how healing the land helps us heal ourselves. It gives real examples like seed-keeping in Tigery and forest care in Congo, to demonstrate how indigenous communities protect the Earth. The hashtags like #EarthDay, #Ecofeminism and #IndigenousLand help people find the post and connect it with global conversations about the environment, women and justice. Another image is a series of slides that contrasts traditional binaries such as man/woman, mind/body, and culture/nature. Each pair is displayed with a slash that is crossed out, creating a strong visual message against this kind of divided thinking, which is often linked to patriarchy. By rejecting these binary divisions, the image supports the ecofeminist idea that we need to think in more inclusive ways. Instead of seeing things as opposites like man versus woman or nature versus culture, ecofeminism encourages us to understand how these elements are linked and interdependent. In addition, a collage made up of photos of tribal women, protest banners, and endangered animals illustrates many issues that ecofeminism deals with colonialism, the loss of animal species, violence against women, and the forced removal of people from their land. The collage brings all these crises together in one powerful image, showing how they are connected. Likewise, an image poses a rhetorical question on a soft, neutral background, prompting viewers to reflect on what ecofeminism asks of them. This simple yet powerful prompt encourages people to move from passive thinking to active responsibility. The image highlights that self-reflection, conversation, and accountability are key parts of practicing ecofeminism, not just something to study. It is something to live through everyday decisions and behaviors. Another post consisting of 12 images highlights the ecofeminist resistance of the Boki Forest Women in Nigeria. It showcases their long-standing struggle against corporate logging and environmental destruction. Through images and texts, it presents women as central figures in defending the forest.

To understand how Facebook is promoting ecofeminism, selected public groups were analyzed based on the relevance of their content. One such public group, consisting of around 638 views, states in its description that ecofeminism celebrates women and nature while examining the intersections of oppression of women, animals, and the environment. The group discusses topics such as feminism, social justice, animal rights, and environmentalism. Most of the content shared are images related to ecofeminism. One post shared on International Women’s Day emphasized how climate change impacts women and girls, citing projections that 158 million more women and girls may face poverty by 2050 and 232 million may face food insecurity. This highlights the group’s engagement with gendered environmental issues. Another group, with over 50 members, focuses primarily on books related to ecofeminism. For instance, in one update, the group announced a discussion of a well-known ecofeminist text scheduled for July 2021, accompanied by a summary of the book’s themes. This reflects the group’s role in promoting ecofeminist ideas through intellectual engagement. Additionally, a third group, consisting of around 40 members, has some updates regarding the ecofeminism theme. The cover image features a broken tree shaped like a girl beneath a dark cloud, evoking themes of destruction, vulnerability, and resistance which are central motifs in ecofeminist thought.

It is observed that groups dedicated entirely to ecofeminist content are very limited in number. The groups discussed earlier include one established in 2013. However, its level of activity is low. The content creation is infrequent, and member engagement is minimal. The other two groups were created after 2020. Their activity is also limited. The posts are very few, and almost no short videos are created and shared. Considering the age and potential of these groups, there is significant room for improvement. To effectively promote ecofeminism, group members need to become more active, by regularly posting, creating, and sharing content that aligns with ecofeminist values and principles. This includes raising awareness about the interconnection between environmental issues and gender justice, as well as highlighting initiatives that protect both nature and women. Only through sustained participation and meaningful content creation can the goals of these groups be fulfilled.

Along with public groups, some public community pages were also explored and analyzed based on their engagement with ecofeminist themes and types of content. It is observed that, compared to the groups, the activity and engagement levels of these community pages are more impressive. One such page with 1,800 likes and 1,800 followers defines ecofeminism as the belief that the oppression of women and the destruction of nature are closely connected. This page regularly shares content from other accounts that align with feminism, ecofeminism, environmental justice, and women’s empowerment. The page has updates on environmental campaigns, events, and activities. Examples include a petition titled “Value Nature as a Living Being, Say Yes,” and another with the phrase “Every signature is a love letter to Earth.” Other posts share updates on global events like a women’s assembly for climate justice, reflecting the page’s focus on awareness-raising through curated material. Another community page, with 1,200 likes and 1,200 followers, presents ecofeminism through both informational and symbolic lenses. It frequently shares content from other sources, such as an article on a woman growing and distributing heirloom seeds to preserve biodiversity, and another about indigenous women advocating for renewable energy. A particularly notable post under the hashtag #WomenFeedTheWorld highlights gender disparities in global agriculture and includes statistics from a prominent institute, emphasizing women’s underrepresentation in land ownership and agricultural services. The page also includes solidarity content supporting indigenous and environmental movements, featuring quotes, photos, and videos focused on environmental activism led by women. Prominent ecofeminist voices are regularly referenced in quotes and shared articles.

Furthermore, another page, with 1,300 likes and 1,400 followers, presents itself as a platform for co-creating a paradigm shift toward a sustainable and climate-resilient future. Its content includes videos of women singing empowerment songs, such as one with lyrics meaning “you take on some of us, you take on all of us—women were born to rise.” One post features a woman holding a placard reading “Women Reclaim Earth,” while another shows a girl walking a tightrope surrounded by natural elements, visually representing the balance between human agency and ecological harmony. The cover photo features a sculpture from England—a reclining woman figure formed from earth, moss, and ivy symbolizes the embodiment of women as part of nature. Additional posts use visual storytelling to express emotional and symbolic connections between women’s autonomy and ecological resistance, such as a silhouette of a woman embracing the horizon at sunset, blending into the landscape as a metaphor for unity with the earth. Accompanied by reflective text, such imagery portrays women reclaiming both personal and planetary freedom from patriarchal domination.

Another community page, with around 700 likes and 748 followers, defines ecofeminism as a movement connecting the exploitation and degradation of the natural world with the oppression of women. Although currently inactive, its past posts include poetry, articles, and various types of content aligned with ecofeminist themes. The visual elements strongly reflect symbolic ecofeminist imagery. Its cover photo features “wood nymphs” blending into a natural landscape, and the profile picture portrays a tree shaped like a woman in a meditative pose, hands folded in prayer symbolizing reverence for nature and the feminine. One particularly evocative post features the image of a pregnant woman whose body is artistically merged with the Earth, her baby bump depicted as the planet itself. This visual metaphor powerfully illustrates the interconnectedness of women and the environment, a core principle of ecofeminism.

## Conclusion

This study investigates the role of three major social media platforms, such as YouTube, Instagram, and Facebook in promoting and communicating the principles of ecofeminism. The content on ecofeminism principles on Youtube includes academic lectures, visual essays, poetic documentaries, and interviews that help audiences engage with ecofeminist theories. Instagram emerges as a vibrant visual space for ecofeminist communication, featuring reels, carousel posts, infographics, and photo essays that use symbolic imagery and targeted hashtags. Posts by environmentalists, educators, and creatives reflect a growing awareness of ecofeminist values on Instagram. In comparison, Facebook shows limited ecofeminist activity. A few public groups and pages engage with ecofeminist ideas through shared articles, event announcements, and discussions. Across all three platforms, ecofeminism is represented more through individual and fragmented efforts rather than institutional and coordinated channels. Audience interaction and regular updates on ecofeminist themes remain minimal. In light of our findings, we recommend some steps to improve the visibility and impact of ecofeminist content on social media. First, more dedicated accounts, channels, and community pages solely focused on ecofeminism are required. Regular and consistent content uploads are essential. Content creators should create varied formats such as reels, tutorials, infographics, and documentaries to suit diverse audience interests. Using features such as relevant hashtags, polls, Q&A sessions, and comment prompts can enhance visibility and encourage user engagement.

The research is limited to only three social media platforms such as Instagram, YouTube, and Facebook. Including other platforms such as Twitter and LinkedIn could provide a broader picture of ecofeminist content on social media. The analysis is based solely on publicly available content, excluding private groups, restricted posts, or non-English material, which may narrow the scope. Future studies could examine various social media platforms to explore their contribution to promote ideals of ecofeminism. Exploring regional languages and locally rooted content would also help explore grassroots ecofeminist expressions.

## Ethics and consent

Ethical approval and consent were not required.

## Data Availability

The data used in this study has been collected from publicly available Instagram, Facebook, and YouTube pages for the purpose of analyzing themes related to ideas and principles of ecofeminism. Due to ethical considerations, the data has been de-identified.
