# Supplementary material for: Women and nature: Ecofeminist study in social media
Source: F1000Res. 2025 Jul 16;14:455. Originally published 2025 Apr 23. [Version 2] doi: 10.12688/f1000research.162436.2 (PMC12381554; doi:10.12688/f1000research.162436.2)
Supplement: Peer review history

# Reviewer report: Reviewer response for version 2

**DOI:** 10.5256/f1000research.183686.r405103

**Published:** 2025-08-26

**Authors:** Kubra Celik

**Affiliations:** 1 Ankara Yıldırım Beyazıt University, Ankara, Turkey

The introductory section is well-designed, moving from general background to the specific focus of the study. It situates ecofeminism within broader discussions of feminism and ecocriticism, referencing significant prior work before clearly stating the rationale for the research. The arguments are logically connected, and the flow of ideas is clear and coherent throughout.

 The methodology is appropriately justified. The authors clearly explain their reasons for selecting the platforms under study and provide sufficient detail about the data collection procedures. Clarifying the time frame of data collection is a strength, as it increases transparency. The use of a flowchart is particularly effective, as it improves readability and helps the reader follow the research design.

 For the results and discussion sections, the paper would benefit from a short introductory paragraph that provides a smooth transition from the methodology and frames the analysis more clearly.

 In terms of expression, the sentence *“Ecofeminism, as a recognized literary theory, is included in the syllabus of various literature, gender studies, and environmental humanities courses”* could be improved by clarifying the word *“various”* with specific examples or references (e.g., *see…*). Furthermore, although the analysis offers concise and relevant insights into ecofeminist themes in media, the academic tone would be strengthened by specifying the media content more precisely. Rather than referring to *“one tutorial, some other videos, or another tutorial,”* the authors could provide direct links, include them as supplementary materials, or describe them in more academically rigorous terms.

 Overall, this article makes a meaningful contribution to the literature on ecofeminism and media studies. With minor revisions to improve clarity and precision, it has the potential to be a strong and valuable publication.

Is the work clearly and accurately presented and does it cite the current literature?

Yes

If applicable, is the statistical analysis and its interpretation appropriate?

Yes

Are all the source data underlying the results available to ensure full reproducibility?

Partly

Is the study design appropriate and is the work technically sound?

Yes

Are the conclusions drawn adequately supported by the results?

Yes

Are sufficient details of methods and analysis provided to allow replication by others?

Yes

Reviewer Expertise:

literary translation, gender studies, feminist translation, ecofeminism, ecocriticism, intertextuality, semiotics of translation

I confirm that I have read this submission and believe that I have an appropriate level of expertise to confirm that it is of an acceptable scientific standard.

# Reviewer report: Reviewer response for version 2

**DOI:** 10.5256/f1000research.183686.r398662

**Published:** 2025-07-17

**Authors:** Graciela Padilla-Castillo

**Affiliations:** 1 Universidad Complutense de Madrid (Spain), Madrid, Spain

I hereby approve the changes made to the document and would like to sincerely thank you for taking my suggestions into consideration. I truly appreciate the effort and attention to detail shown in incorporating the proposed amendments, as well as the openness to dialogue throughout this process.

Is the work clearly and accurately presented and does it cite the current literature?

Yes

If applicable, is the statistical analysis and its interpretation appropriate?

Yes

Are all the source data underlying the results available to ensure full reproducibility?

Yes

Is the study design appropriate and is the work technically sound?

Yes

Are the conclusions drawn adequately supported by the results?

Yes

Are sufficient details of methods and analysis provided to allow replication by others?

Yes

Reviewer Expertise:

Information theory, ethics, and deontology of communication, TV fiction, gender studies, social media, journalism, feminist studies.

I confirm that I have read this submission and believe that I have an appropriate level of expertise to confirm that it is of an acceptable scientific standard.

# Reviewer report: Reviewer response for version 1

**DOI:** 10.5256/f1000research.178637.r383136

**Published:** 2025-05-26

**Authors:** Ignacio-Jesús Serrano-Contreras

**Affiliations:** 1 Warsaw University, Warszawa, Poland

First, I am grateful for the interest in the review that I can offer. Next, I believe that this is an interesting topic that needs to be addressed in depth.

 1. The methodological design seems to me to be somewhat vague. The data collection and identification were not explained in depth. In this case, it is appropriate to ask what kind of methods, software, or libraries used. At the same time, it is not clear what content is considered relevant, which makes reproducibility difficult.

 2. Importantly, the time frame of the study is not clearly indicated since both as a study variable and as a criterion of understanding, it is necessary to know this question in a more substantial and explanatory way.

 3. Doubts are generated regarding the type of sample because the content creators or the type of identification used for the analysis and collection are not made clear.

 4. As far as data analysis is concerned, qualitative content analysis does not specify which techniques are followed and whether we are talking about software, coding schemes or how consistency was ensured.

 5. It is not clear how the type of data and the mention of this multi-method model are integrated. Without a solid explanation, the mixed approach seems more like a generic statement than a well-defined strategy.

 6. Questions arise as to how the brains have been addressed and whether, for example, triangulation (such as the incorporation of independent/external perspectives or reviews) has been executed.

 7. The claim to have an objective mindset is doubtful because the research is primarily qualitative and is based on an interpretative process.

 8. Lack of explanation in the selection of social networks and whether that might imply an explanatory variable for this and other work are not explained.

 9. The method section includes an extensive discussion of ecofeminist theory, which, although relevant, would be out of place in the above section.

 Although these parts need to be clarified and corrected, we provide a brief explanation of how to proceed:

- Specifying the sample collection process
- Detailed data analysis
- Strengthen ethical considerations
- Justifying the selected platforms
- Reorganizing the content

Mostly, the considerations are methodological in nature, as this is the basis that must underpin any scientific work in order to provide a clear exposition and a reflection and discussion that will continue to provide knowledge.

Is the work clearly and accurately presented and does it cite the current literature?

Partly

If applicable, is the statistical analysis and its interpretation appropriate?

No

Are all the source data underlying the results available to ensure full reproducibility?

No

Is the study design appropriate and is the work technically sound?

Partly

Are the conclusions drawn adequately supported by the results?

No

Are sufficient details of methods and analysis provided to allow replication by others?

No

Reviewer Expertise:

Methodology

I confirm that I have read this submission and believe that I have an appropriate level of expertise to confirm that it is of an acceptable scientific standard, however I have significant reservations, as outlined above.

# Reviewer report: Reviewer response for version 1

**DOI:** 10.5256/f1000research.178637.r383138

**Published:** 2025-05-14

**Authors:** Graciela Padilla-Castillo

**Affiliations:** 1 Universidad Complutense de Madrid (Spain), Madrid, Spain

While the article is strong in all major areas, the following minor improvements could further enhance its clarity and impact:

 - For absolute clarity, consider including a flowchart or diagram summarizing the study design, especially if the methodology is complex. This can help readers quickly grasp the experimental workflow.

 - Although limitations are acknowledged, a more detailed discussion of how these might affect the interpretation of results would strengthen the manuscript. For instance, address any potential confounding variables or biases that could influence the findings. 

 - The discussion could be expanded to more explicitly address the broader implications of the findings for the field and suggest concrete directions for future research.

Is the work clearly and accurately presented and does it cite the current literature?

Yes

If applicable, is the statistical analysis and its interpretation appropriate?

Yes

Are all the source data underlying the results available to ensure full reproducibility?

Yes

Is the study design appropriate and is the work technically sound?

Yes

Are the conclusions drawn adequately supported by the results?

Yes

Are sufficient details of methods and analysis provided to allow replication by others?

Yes

Reviewer Expertise:

Information theory, ethics, and deontology of communication, TV fiction, gender studies, social media, journalism, feminist studies.

I confirm that I have read this submission and believe that I have an appropriate level of expertise to confirm that it is of an acceptable scientific standard.
